# Atlas Toolkit: Fast registration of 3D morphological datasets in the absence of landmarks

**DOI:** 10.1038/srep20732

**Published:** 2016-02-11

**Authors:** Timothy Grocott, Paul Thomas, Andrea E. Münsterberg

**Affiliations:** 1 School of Biological Sciences, University of East Anglia, Norwich Research Park, Norwich, NR4 7TJ, U.K

## Abstract

Image registration is a gateway technology for Developmental Systems Biology, enabling computational analysis of related datasets within a shared coordinate system. Many registration tools rely on landmarks to ensure that datasets are correctly aligned; yet suitable landmarks are not present in many datasets. Atlas Toolkit is a Fiji/ImageJ plugin collection offering elastic group-wise registration of 3D morphological datasets, guided by segmentation of the interesting morphology. We demonstrate the method by combinatorial mapping of cell signalling events in the developing eyes of chick embryos, and use the integrated datasets to predictively enumerate Gene Regulatory Network states.

Imaging combined with computational analysis is an important tool for illuminating organogenesis in the embryo^1^. Typically in fluorescence microscopy studies, it is possible to observe a maximum of three or four molecular species in a single embryo specimen, depending on the availability of orthogonal fluorescent probes. There is a need therefore to develop broadly applicable tools for integrating three- and four-dimensional (3D & 4D) imaging datasets from multiple specimens^2^. Image registration provides a means of mapping diverse observations from independent embryo specimens into a unified coordinate system, thereby permitting system-level analysis.

Registration is often performed in pair-wise fashion^3^,^4^, with multiple images being registered to a pre-selected reference specimen. For landmark-rich objects such as whole embryos, user-defined fiduciary markers identifying corresponding features may be employed to guide the process^3^,^4^. In other cases, landmarks may be identified by molecular labelling, e.g. to reveal neuropil morphology in brain specimens^5^. Unfortunately, datasets from isolated tissues and organ primordia such as the developing eye offer few or no well-defined landmarks, making the placement of fiduciary markers impossible. Moreover, an over-reliance on molecular markers will also consume precious data channels, limiting the information that may be captured per specimen.

We have developed an accessible, and generally applicable, software toolkit to achieve efficient landmark-free group-wise registration of corresponding objects segmented from 3D imaging datasets, such that objects converge towards their consensus morphology.

## Results & Discussion

### An efficient method for 3D group-wise elastic registration without landmarks

**Figure 1:**
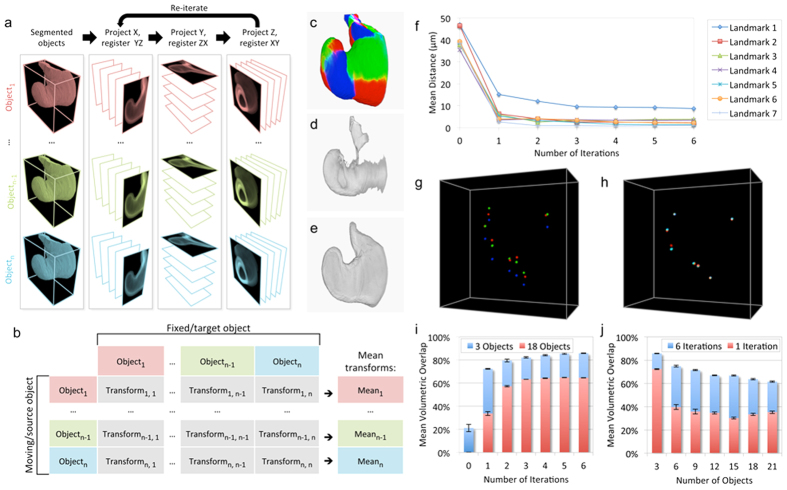
Group-wise 3D object registration using Atlas Toolkit. **(a)** Group-wise registration of multiple 3D objects is achieved by the sequential and iterative registration of orthogonal 2D projections. **(b)** For each orthogonal plane, the 2D projections are registered group-wise by determining the mean transformation coefficients resulting from multiple pair-wise registrations. **(c)** 3D reconstruction of three superimposed Optic Vesicle objects (red, green and blue, respectively) segmented from stage HH12 chick embryos before group-wise 3D registration. **(d)** Intersection of the three Optic Vesicle objects shown in (**c**) before group-wise 3D registration. **(e)** Intersection of the three Optic Vesicle objects shown in (**c**) after group-wise 3D registration using Atlas Toolkit. **(f)** Graph quantifying the convergence of 7 simulated landmarks in three manually deformed objects, expressed as mean pair-wise distance in microns (μm), with increasing iteration numbers. **(g)** 3D reconstruction showing the initial distribution of 7 simulated landmarks in the three manually deformed objects (red, green, blue). **(h)** 3D reconstruction showing the convergence of landmarks in (**g**) following registration using Atlas Toolkit. **(i)** Relationship between mean volumetric overlap and increasing iteration number, for three or 18 real Optic Vesicle objects. **(j)** Relationship between mean volumetric overlap and increasing numbers of real Optic Vesicle objects, for one or six iterations.

Atlas Toolkit (Supplementary Software 1) is a collection of Fiji/ImageJ plugins^6^ developed to achieve group-wise elastic registration of 3D (XYZ) objects by decomposing the alignment problem into a sequence of orthogonal 2D elastic registrations (e.g. YZ > ZX > XY; Fig. 1a), for which each object is registered to every other. The method is guided by the gross morphology of the corresponding 3D objects, which in our study are the Optic Vesicles of developing eyes. The tool “*Label Registration 3D*” accepts a group of *n* pre-segmented objects (“.label” files), each created using the Fiji/ImageJ^6^ Segmentation Editor. Beginning with the first of the three orthogonal planes (e.g. YZ), it generates a 2D average intensity projection along the third axis (e.g X) for each object in the group. An existing 2D registration algorithm (bUnwarpJ^7^) then performs consistent and elastic pair-wise registration for each object-pair in the group (Fig. 1b), yielding *n* sets of 2D transformation coefficients for each object. 2D group-wise registration is achieved within the current plane by firstly, calculating the set of mean 2D transformation coefficients for each object (Fig. 1b), and then applying this mean transformation to all 2D slices in that object’s image stack. This 2D group-wise registration is then repeated for the two remaining orthogonal planes (e.g. ZX, XY; Fig. 1a) such that all objects in the group converge towards their consensus 3D morphology. The registration process may be performed iteratively until an optimal alignment is achieved.

Figure 1c shows the merged 3D reconstructions of three Optic Vesicle objects (red, green and blue, respectively) segmented from independent 3D datasets of stage HH12^8^ chick embryos, prior to their registration (Supplementary Video 1). The extent of their misalignment, due to small variations in morphology, is revealed by their broken intersection (Fig. 1d; Supplementary Video 2). Following registration using Atlas Toolkit, the three Optic Vesicle objects have converged together as indicated by their improved intersection (Fig. 1e; Supplementary Video 3), which we take to be the consensus morphology for this group of tissues. In addition, the registration process also yields an ‘orthogonal transform sequence’ (“.ots” file) for each object, which describes its sequential transformation. This is used in conjunction with another tool, “*Apply Label Registration*”, to transform the original or derivative dataset(s) towards the consensus morphology, facilitating comparative analyses of the underlying 3D datasets within a shared coordinate system.

### Performance evaluation using simulated datasets

To aid comparison with other 3D registration tools, we first evaluated our method using an artificial dataset consisting of three manually deformed Optic Vesicle objects, each derived from a single Optic Vesicle tissue to which seven simulated landmarks had been added (see Supplementary Methods). The simulated dataset underwent registration with between one and six iterations and the resulting alignments were evaluated using two measures: 1) the mean Euclidean distance between corresponding landmark-pairs measured in microns (μm), and 2) the mean volumetric overlap between all registered objects expressed as a percentage of total volume. Figure 1f quantifies the mean pair-wise distance for all seven converging landmarks. Following six iterations, the mean distance between all 21 landmark-pairs (seven landmarks x three object-pairs) was 3.24 μm ± 3.6 μm (mean ± standard deviation), while the mean volumetric overlap was 95.9% ± 0.14% (mean ± standard deviation). It may be of interest to note that this average registration error is less than one nuclear diameter, although nuclear positioning is not expected to correspond between specimens due to inter-kinetic nuclear migration and natural variation in cell numbers. The 3D renderings in Fig. 1g,h show the distribution of the seven simulated landmarks in all three objects (red, green and blue), before and after registration respectively.

### Comparison with other freely available 3D registration tools

Although Atlas Toolkit is intended to register datasets for which landmarks cannot be defined, it is informative to directly compare its performance to that of an accurate landmark-based method. This comparison is possible because our synthetic dataset includes simulated landmarks. BrainAligner^5^ (http://penglab.janelia.org/proj/brainaligner/) employs user-defined landmarks present in each of the objects to produce accurate 3D elastic registrations in pair-wise fashion. When supplied with precise coordinates for simulated landmarks in all three synthetic datasets (described above), BrainAligner succeeds in registering those landmarks to an average distance of 0.4 μm ± 0.31 μm (mean ± standard deviation; BrainAligner ‘Method A’ in Supp. Fig. S1a), giving a volumetric overlap of 98.8% ± 0.3% (mean ± standard deviation; Supp. Fig. S1b).

In addition to this positive benchmark, we also deliberately misuse BrainAligner to demonstrate the fail-case in which a landmark-based method is inappropriately used to register a landmark-deficient dataset. In this case landmark coordinates were supplied for only one of the three objects, forcing BrainAligner to attempt auto-detection of corresponding landmarks in the other two. This simulates the situation in which a user inappropriately defines arbitrary ‘landmarks’ for a landmark-deficient dataset in an attempt to force an alignment. As might be expected, this second run failed to yield an accurate alignment: the registration error increased 60-fold to 24.27 μm ± 15.51 μm (mean ± standard deviation; BrainAligner ‘Method B’ in Supp. Fig. S1a) and the volumetric overlap was only 62.98% ± 9.72% (mean ± standard deviation; Supp. Fig. S1b). This inappropriate use-case highlights both the importance of precisely locating true landmarks and the difficulty of forcing such landmark-based methods to register landmark-deficient objects.

The Computational Morphometry Toolkit^9^ (CMTK; http://www.nitrc.org/projects/cmtk) includes a landmark-independent method for elastic group-wise registration of 3D datasets (groupwise_warp). We evaluated its performance using the same artificial dataset to provide a more direct performance comparison with our landmark-free method. CMTK produced a registration error of 10.85 μm ± 9.35 μm (mean ± standard deviation; Supp. Fig. 1a), which is around 3-fold higher than our method. However, CMTK yielded a superior volumetric overlap of 99.52% ± 0.02% (mean ± standard deviation; Supp. Fig. 1b). A more in-depth discussion of these results is included in Supplementary Information.

Atlas Toolkit and BrainAligner registered the pre-segmented datasets in a matter of minutes using a laptop computer, whereas CMTK took nearly two days and required a workstation computer due to its greater demand for random access memory. Total registration times, including manual segmentation time for landmark-free methods (Atlas Toolkit and CMTK), are shown in Supplementary Figure S1c.

### Impact of group size and iteration number with real datasets

We next evaluated the effect of both iteration number and group size (object number) when registering real landmark-free objects, with mean volumetric overlap being the only measure available. Figure 1i highlights an improvement of alignment quality with additional iterations, but shows that the degree of improvement diminishes with successive iterations. The larger group size (18 objects versus three) benefits most from additional iterations, but the degree of improvement plateaus more quickly. Correspondingly, Fig. 1j shows how alignment quality may suffer as object number increases, but also that the initially high penalty (increasing from three to six objects) observed in this case can be minimised by performing more iterations.

### Using Atlas Toolkit to map combinatorial cell signalling in the Optic Vesicle

**Figure 2:**
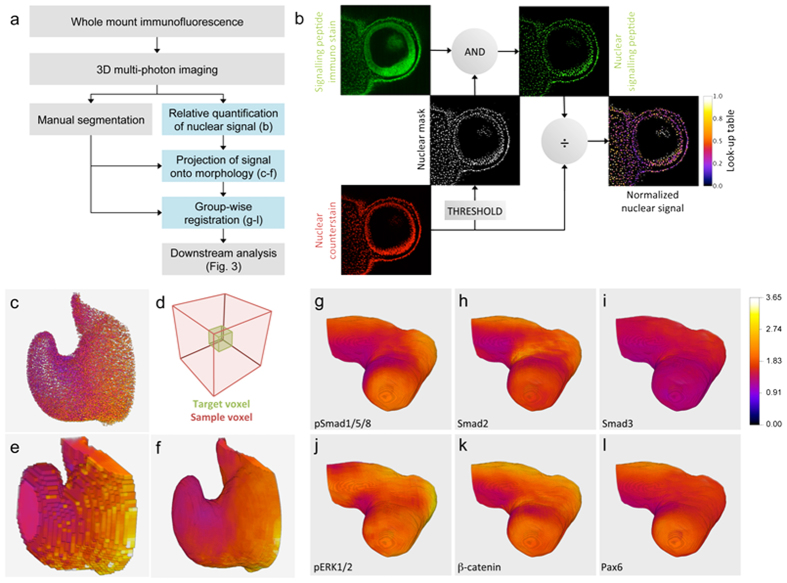
Application of Atlas Toolkit to mapping nuclear protein levels against Optic Vesicle morphology for eighteen stage HH10 chick embryos. **(a)** Overview of method workflow with blue boxes highlighting the steps performed by Atlas Toolkit. **(b)** Method for relative quantification of nuclear protein levels used by the tool ‘Extract Nuclear Signal’. **(c–f)** Method for projecting nuclear signal onto surface morphology used by the tool ‘Project to Segment Label’: **(c)** 3D reconstruction of cell nuclei segmented from an HH12 Optic Vesicle, colour-coded according to phospho-ERK1/2 protein levels (cold colours = low signal; warm colours = high signal). **(d)** Local averaging is used to ‘fill-in’ intra-nuclear space: the image volume is sub-divided into 12 μm target voxels, each of which is assigned the mean nuclear signal level from its surrounding 36 μm ‘sample voxel’. **(e)** Result of local averaging applied to cell nuclei in **(c)**.**(f)** Surface morphology is restored by cropping the locally averaged volume in (**e**) using the segmentation label. **(g–l)** Group-wise 3D registration of Optic Vesicles segmented from 18 stage HH10 chick embryos stained with six different antibodies (three embryos per antibody). The nuclear signal levels were quantified and projected onto Optic Vesicle morphology prior to registration. The Optic Vesicle morphology shown in (**g–l**) is the consensus of all 18 embryos, while each antibody stain is the mean average of three embryos (normalised to background): **(g)** nuclear phospho-Smad1/5/8 (n = 3); **(h)** nuclear Smad2 (n = 3); **(i)** nuclear Smad3 (n = 3); **(j)** nuclear phospho-ERK1/2 (n = 3); **(k)** nuclear β-catenin (n = 3); **(l)** nuclear Pax6 (n = 3).

We applied Atlas Toolkit to the problem of mapping cell-signalling events in the Optic Vesicle of the developing eye (method summarised in Fig. 2a; see Online Methods). Briefly, individual stage HH10 chick embryos^8^ underwent whole-mount immunofluorescence labelling for one of five intracellular signalling proteins (phospho-Smad1/5/8; Smad2; Smad3; phospho-ERK1/2; β-catenin) or a downstream eye transcription factor (Pax6). Embryos were optically cleared^10^, and cell nuclei counterstained in preparation for two-photon optical sectioning of the developing eye. The active signalling status of the observed proteins was established by measuring their nuclear fluorescence signals relative to the nuclear counter-stain with the tool “*Extract Nuclear Signal*” (Fig. 2b) analogous to an earlier study of 2D histological sections^11^. In a parallel step, Optic Vesicle objects were segmented using the Fiji/ImageJ Segmentation Editor (Fig. 2c), and the relative nuclear fluorescence of each protein was then projected via local averaging (Fig. 2d) onto the segmented object using the tool “*Project to Segment Label*” (Fig. 2e,f).

Three embryos were labelled for each of the six proteins (five signalling molecules, one transcription factor), yielding a total of 18 3D datasets. Segmented Optic Vesicle objects from all 18 datasets where registered together using the tool “*Label Registration 3D*”, to yield the consensus Optic Vesicle morphology for this group. The three morphological projections for each of the six proteins were then transformed into the shared coordinate system of the consensus morphology using the tool “*Apply Label Registration*”, and mean distributions for each of the six proteins were then determined using the “*Merge Registered Volumes*” tool. Figure 2g–l reconstructs the mean distributions for all six proteins, each mapped against the consensus Optic Vesicle morphology.

### Atlas Toolkit as a gateway to systems analyses: predicting GRN states

**Figure 3:**
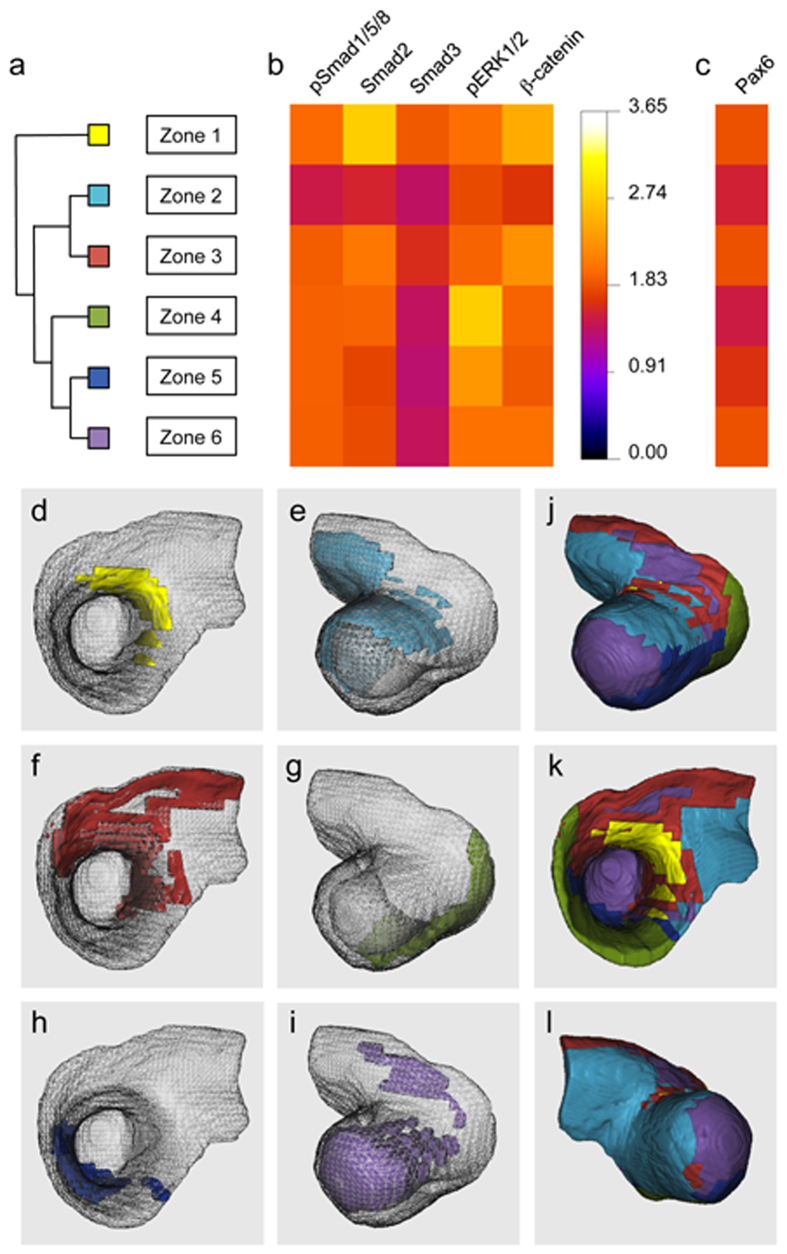
Hierarchical clustering of ‘Optic Vesicle-space’ reveals multiple domains of divergent combinatorial cell signalling, and predicts spatially distributed Gene Regulatory Network states. **(a)** Dendrogram showing the hierarchical relationship of six Optic Vesicle zones with divergent signalling profiles. **(b)** Heat map showing the mean nuclear signal levels (normalised to background) for each of the identified clusters/zones. **(c)** Heat map showing mean nuclear Pax6 level (normalised to background) for each cluster/zone. **(d–i)** 3D reconstruction of each of the six zones shown within the consensus Optic Vesicle morphology. **(d)** Zone 1, apical/medial view. **(e)** Zone 2, basal/dorsal-lateral view. **(f)** Zone 3, apical/medial view. **(g)** Zone 4, basal/dorsal-lateral view. **(h)** Zone 5, apical/medial view. **(i)** Zone 6, basal/dorsal-lateral view. **(j–l)** Three different views of the wholly reconstructed consensus Optic Vesicle morphology, colour-coded to display the six zones in (**d–i**). **(j)** Basal/dorsal-lateral view. **(k)** Apical/medial view. **(l)** Basal/caudal-lateral view.

We reasoned that, since signalling pathway activities drive Gene Regulatory Network (GRN) state transitions, we might be able to predict the physical distribution of GRN states within the Optic Vesicle by hierarchical clustering of ‘Optic Vesicle-space’ according to the five signalling activities observed (Fig. 2g–k). The tool “*Sample Volumes for Clustering*” was used to format the registered datasets (Fig. 2g–k) in preparation for hierarchical clustering using the software Cluster3.0^12^. The clustering results were then imported back into Fiji/ImageJ using the tool “*Cluster Viewer*”, which reconstructs the clustered volume and displays it using the Fiji/ImageJ 3D Viewer function. This revealed morphological zones that exhibit six unique signalling profiles (Fig. 3a,b), and thus predicting six spatially distributed GRN states (Fig. 3d–i). The dendrogram in Fig. 3a shows the relationships of the six zones (presumptive GRN states), while Fig. 3b shows a heat map summarising the mean nuclear signal level within each zone for all five signalling proteins. Figure 3c shows a corresponding heat map for the transcription factor Pax6. 3D reconstructions of each predicted GRN state/zone were created using the “*Cluster Viewer*” tool (Fig. 3d–i), and Fig. 3j–l shows three different views of the reconstructed Optic Vesicle with all six zones displayed. In order to test the predictive power of this approach, the mean distribution of the downstream transcription factor Pax6 was excluded from the clustering analysis. As can be seen, the distribution of Pax6 protein (Fig. 2l) most closely resembles the predicted GRN state corresponding to Zone 6 (Fig. 3i).

In summary, Atlas Toolkit makes accessible a broadly applicable method for landmark-free alignment of 3D imaging datasets. Within experimental fields such as Developmental Biology, a number of barriers have hindered the transition of existing image registration approaches from being a niche technique^3^,^4^ to a more widely adopted tool for system-level analysis. Atlas Toolkit aims to make registration of 3D morphological datasets readily accessible to both experimental and computational biologists alike.

## Methods

### Chick embryos, antibodies & whole-mount immunofluorescence

All animal experiments were conducted in accordance with UK Home Office guidelines. All chick embryos were harvested before half of the incubation period had elapsed and the work is therefore exempt from requiring UK Home Office approval.

Fertile hen’s eggs (Henry Stewart) were incubated at 38 °C to stage HH10^8^. Embryos were harvested into ice-cold PBS and fixed in 4% PFA in PBS for 90 minutes at 4 °C. Heads were isolated, bisected along the midline and dehydrated via methanol series (25%, 50%, 75% in PBS-Tween) before storing overnight in 100% methanol at −20 °C. Following re-hydration, the samples were blocked in PBTS solution (PBS with BSA, Triton X-100 and goat serum) overnight at 4 °C.

Tissues were incubated in primary antibodies, diluted in PBTS at the concentrations indicated in Supplementary Table 1, at 4 °C for 3–5 days. Samples were then incubated with either AlexaFluor488-conjugated (1:500, Molecular Probes A-11034 & A-11001) or Biotin-conjugated (1:200, Vector Labs BA-1000) secondary antibodies diluted in PBTS, overnight at 4 °C. Those specimens incubated with Biotin-conjugated secondary were further incubated with AlexaFluor488-conjugated Streptavidin (1:500, Molecular Probes S-11223) diluted in PBTS at 4 °C overnight.

### Clearing, counter-staining & mounting

Specimens were optically cleared in Scale A2^10^ for two weeks at 4 °C before counter-staining cell nuclei with Propidium Iodide/RNase (Cell Signalling Technology #4087S) overnight at 4 °C. Specimens were mounted in AF1 mounting reagent (Citifluor) and stored at 4 °C overnight.

### Multi-photon microscopy

Two-photon microscopy was performed using a LaVision Biotec TriMScope II instrument with inverted stand and ImSpector Pro software. Typically, a volume of 500 um × 500 um × 250 um was imaged using a 20X air objective with a numerical aperture of 0.8. AlexaFluor488 and Propidium Iodide underwent simultaneous two-photon excitation with a single laser line (Coherent Vision II Ti:Sapphire, pulsed femtosecond laser) at a wavelength of 930 nm and scan frequency of 200 Hz. AlexaFlour488 and Propidium Iodide fluorescence were separated using emission filters at 525+/−25 nm and 620+/−30 nm, respectively, and captured using a pair of sensitive, non-descanned GaAsP detectors. Two-photon image stacks were generated with an anisotropic pixel resolution of 0.333 μm (X) by 0.333 μm (Y) by 0.72 μm (Z).

### Computational analysis

With the exception of hierarchical clustering, all computational analysis was performed in Fiji/ImageJ^6^. Image segmentation employed the integrated Segmentation Editor. All other operations were performed using Atlas Toolkit. See Supplementary Methods for full details.

### Evaluation of registration performance

For synthetic datasets, the mean Euclidean distance between pairs of corresponding landmarks was used as a performance metric together with the mean percentage overlap between each object and the group consensus. For real landmark-free datasets, only the mean percentage overlap between each object and the group consensus was used. See Supplementary Methods for further details. The registration performance of Atlas Toolkit was directly compared with that of BrainAligner^5^ (http://penglab.janelia.org/proj/brainaligner/) and CMTK^9^ (http://www.nitrc.org/projects/cmtk) using the same synthetic dataset.

### Hierarchical clustering

Following registration, the combined dataset was sampled with an isotropic voxel size of 18 μm^3^ using Atlas Toolkit, before hierarchical clustering according to Euclidean distance (complete linkage) using the software Cluster 3.0^12^. Clustering results were visualised in Fiji/ImageJ using Atlas Toolkit. See Supplementary Methods for full details.

### Code availability

The Atlas Toolkit plugin for Fiji/ImageJ is free software. Both the compiled plugin file and source code used in this study are included in Supplementary Software 1, together with sample test data. The latest source code can be accessed at (https://github.com/GrocottLab/Atlas-Toolkit). The latest plugin can be installed via our update site (http://sites.imagej.net/GrocottLab/). A wiki including installation instructions and video demonstrations can be accessed at (http://fiji.sc/Atlas_Toolkit).

## Additional Information

**How to cite this article**: Grocott, T. *et al.* Atlas Toolkit: Fast registration of 3D morphological datasets in the absence of landmarks. *Sci. Rep.* **6**, 20732; doi: 10.1038/srep20732 (2016).

## Supplementary Material

Supplementary Information

Supplementary Video 1

Supplementary Video 2

Supplementary Video 3

Supplementary Video 4

Supplementary Video 5

Supplementary Video 6

Supplementary Software 1

## References

[b1] Megason S. G. & Fraser S. E. Imaging in systems biology. Cell. 130, 784–795 (2007). 17803903 10.1016/j.cell.2007.08.031

[b2] Luengo-Oroz M. A., Ledesma-Carbayo, M. J., Peyriéras, N. & Santos A. Image analysis for understanding embryo development: a bridge from microscopy to biological insights. Curr. Opin. Genet. Dev. 21, 630–637 (2011). 21893410 10.1016/j.gde.2011.08.001

[b3] Armit C. *et al.* eMouseAtlas, EMAGE, and the spatial dimension of the transcriptome. Mamm. Genome 23, 514–524 (2012). 22847374 10.1007/s00335-012-9407-1 PMC3463796

[b4] Fisher M. E. *et al.* Integrating technologies for comparing 3D gene expression domains in the developing chick limb. Dev. Biol. 317, 13–23 (2008). 18355805 10.1016/j.ydbio.2008.01.031 PMC2529376

[b5] Peng H. *et al.* BrainAligner: 3D registration atlases of Drosophila brains. Nat. Methods 8, 493–498 (2011). 21532582 10.1038/nmeth.1602 PMC3104101

[b6] Schindelin J. *et al.* Fiji: an open-source platform for biological-image analysis. Nat. Methods 9, 676–682 (2012). 22743772 10.1038/nmeth.2019 PMC3855844

[b7] Arganda-Carreras I. *et al.* Consistent and elastic registration of histological sections using vector-spline regularization. Lecture Notes in Computer Science 4241, 85–95 (Springer, 2006).

[b8] Hamburger V. & Hamilton H. L. A. series of normal stages in the development of the chick embryo. J. Morph. 88, 49–92 (1951). 24539719

[b9] Rohlfing T. & Maurer C. R. J. Nonrigid image registration in shared-memory multiprocessor environments with application to brains, breasts, and bees. IEEE Trans. Inf. Technol. Biomed. 7, 16–25 (2003). 12670015 10.1109/titb.2003.808506

[b10] Hama H. *et al.* Scale: a chemical approach for fluorescence imaging and reconstruction of transparent mouse brain. Nat. Neurosci. 14, 1481–1488 (2011). 21878933 10.1038/nn.2928

[b11] Schohl A. & Fagotto F. Beta-catenin, MAPK and Smad signaling during early Xenopus development. Development 129, 37–52 (2002). 11782399 10.1242/dev.129.1.37

[b12] de Hoon M. J., Imoto S., Nolan J. & Miyano S. Open source clustering software. Bioinformatics 20, 1453–1454 (2004). 14871861 10.1093/bioinformatics/bth078

