# Supplementary material for: Atlas Toolkit: Fast registration of 3D morphological datasets in the absence of landmarks
Source: Sci Rep. 2016 Feb 11;6:20732. doi: 10.1038/srep20732 (PMC4749973; doi:10.1038/srep20732)
Supplement: Supplementary Information [file srep20732-s1.pdf]

Atlas Toolkit: Fast registration of 3D morphological datasets in the absence of landmarks

Timothy Grocott[1*], Paul Thomas[1], Andrea E Münsterberg[1]

1. School of Biological Sciences, University of East Anglia, Norwich Research Park, Norwich, NR4 7TJ, U.K.

* Corresponding Author: t.grocott@uea.ac.uk

Supplementary Methods

1. Immuno labelling of signalling proteins in whole-mount chick embryos.

Fertile hen's eggs (Henry Stewart) were incubated at 38 ºC for approximately 36 hours until stage HH10[1]. Embryos were harvested into ice-cold PBS and fixed in 4 % PFA in PBS for 90 minutes at 4 ºC. Following fixation, embryos were washed in PBS-Tween and their heads were isolated, bisected along the midline and dehydrated by passing through a methanol series (25 %, 50 %, 75 % in PBS-Tween) before storing overnight in 100 % methanol at -20 ºC. The tissues were then re-hydrated through a methanol series (75 %, 50 %, 25 % in PBS-Tween), washed in PBS-Tween and blocked in PBTS solution (BSA, Triton X-100 and goat serum) overnight at 4 ºC.

Tissues were incubated in primary antibodies, diluted in PBTS at the concentrations indicated in Supplementary Table 1, at 4 ºC for 3 – 5 days. Following extensive washing in PBS-Tween (three brief washes and eight extended washes of one hour each at 4 ºC), tissues were incubated with either AlexaFluor488-conjugated (1:500, Molecular Probes A-11034 & A-11001) or Biotin-conjugated (1:200, Vector Labs BA-1000) secondary antibodies diluted in PBTS, overnight at 4 ºC, followed by further extensive washing. Specimens incubated with Biotin-conjugated secondary were further incubated with AlexaFluor488-conjugated Streptavidin (1:500, Molecular Probes S-11223) diluted in PBTS at 4 ºC overnight.

The specimens were optically cleared by passing through 25 %, 50 %, 75 % and 100 % Scale A2 clearing solution[2], and incubated in 100 % Scale A2 for two weeks at 4 ºC. Once cleared, specimens were returned to PBS-Tween via 75 %, 50 % and 25 % Scale A2, and incubated with a solution of Propidium Iodide/RNase (Cell Signalling Technology) overnight at

4 ºC to counter-stain cell nuclei. Reversing the sequence of Scale A2 > Propidium Iodide causes the latter to be washed from the specimen whereas antibody labelling remains intact. Specimens were extensively washed in PBS-Tween and passed through a series of 25 %, 50 %, 75 % and 100 % AF1 mounting reagent (Citifluor).

Each specimen was mounted for microscopy by punching a hole through three or four layers of electrical tape before adhering to a clean glass microscope slide. A specimen was pipetted into the resulting chamber, ensuring that the level of AF1 mounting reagent protruded slightly above the surrounding electrical tape. A clean rectangular cover glass was then applied over the top, ensuring that air bubbles were excluded, and secured in place with nail polish. Once mounted, specimens were stored inverted at 4 ºC overnight.

Supplementary Table 1 | Primary antibodies used for whole-mount immuno labelling

**Antigen:**	**Source:**	**Cat. #:**	**Dilution:**
Phospho Smad1 (Ser463/465) / Smad5 (Ser463/463) / Smad8 (Ser426/428)	Cell Signaling Technology	#9511	1:100
Total Smad2	Cell Signaling Technology	#3122	1:400
Total Smad3	Cell Signaling Technology	#9523	1:100
Phospho p44/p42 (Erk1/2) (Thr202/Tyr204)	Cell Signaling Technology	#4370	1:200
Total β-catenin	Cell Signaling Technology	#2677	1:200
Pax6	Developmental Studies Hybridoma Bank	PAX6	1:50

2. Two-photon microscopy of whole-mount chick embryos.

Two-photon microscopy was performed using a La Vision Biotech TriMScope II instrument with inverted stand and ImSpector Pro software. Typically, a volume of 500 um x 500 um x 250 um was imaged using a 20X air objective with a numerical aperture of 0.8. AlexaFluor488 and Propidium Iodide underwent simultaneous two-photon excitation with a single laser line (Coherent Vision II Ti:Sapphire, pulsed femtosecond laser) at a wavelength of 930 nm and scan frequency of 200 Hz. AlexaFlour488 and Propidium Iodide fluorescence were separated using emission filters at 525 +/- 25 nm and 620 +/- 30 nm, respectively, and captured using a pair of sensitive, non-descanned GaAsP detectors. Two-photon image stacks were generated at an oversampled resolution of 0.333 µm (X) by 0.333 µm (Y) by 0.72 µm (Z). The resulting datasets were exported as OME Tiff files for subsequent analysis using the Fiji distribution of ImageJ[3].

3. Relative quantification of nuclear protein levels in Fiji/ImageJ.

"Atlas Toolkit > 1. Extract Nuclear Signal"

To account for variable depth penetration and shadowing across the complex tissue morphology of the 3D specimens, signalling protein levels indicated by AlexaFlour488 fluorescent signal was normalised to that of the Propidium Iodide nuclear counter stain. Moreover, since the signalling proteins under investigation translocate the cell nucleus upon activation, the Propidium Iodide fluorescent signal was also used to isolate nuclear fluorescence as has been performed previously[4].

This was necessary for Smad2, Smad3 and β-catenin proteins, for which the antibodies utilised do not report phosphorylation status. It is also necessary for the utilised phospho-Smad1/5/8 antibody since phosphatase treatment of specimens revealed that this antibody strongly labelled an unidentified and non-phosphorylated epitope in the cytoplasm of mitotic cells, in addition to the intended nuclear phosphorylated-Smad1/5/8 signal.

The Atlas Toolkit includes a tool "1. Extract Nuclear Signal" that recapitulates the standardised method used for quantifying nuclear AlexaFlour488 fluorescence relative to that of Propidium Iodide in this study. This method may need to be adjusted for different biological specimens and imaging conditions.

The method for relative quantification is schematised in Figure 2b. The Propidium Iodide signal (channel 1) was subjected to Auto-Local Thresholding in Fiji/ImageJ in order to generate a binary mask were the locations of cell nuclei were represented with a pixel value of 255 (white), where as non-nuclear pixels had a value of zero (black). The resulting 8-bit stack was converted back to 16-bit (Image > Type > 16-bit) and divided by 255 (Process > Math > Divide…) such that nuclear pixels were represented by a numerical value of 1. The resulting 16-bit pixel mask was saved and the original 16-bit Propidium Iodide (PI, channel 1) image stack re-opened. These two stacks (nuclear mask and PI channel 1) were multiplied (Process > Image Calculator…) to isolate the nuclear fraction of PI channel 1. The original 16-bit AlexaFluor488 (channel 2) image stack was then opened and also multiplied by the nuclear mask. Thus, non-nuclear pixels in channels 1 & 2 have a value of zero, whereas nuclear pixels have a value corresponding to the observed nuclear fluorescence signal.

To determine the relative nuclear fluorescence of AlexaFluor488 normalised to the PI counter-staining, channel 2 was then divided by channel 1 (Process > Image Calculator...,

‘32-bit (float) result’ option checked). Since non-nuclear pixels are divided by zero, it is important to set Fiji/ImageJ to fill these pixels with a “divide by zero value” of zero instead of the default value of infinity or NaN (‘Not a Number’). This option should be set (Edit > Options > Misc…) before running the Image Calculator. The resulting 32-bit image stack represents the normalised indirect nuclear fluorescence signal for the protein of interest, and can more readily be visualised by applying a colour map or lookup table to the stack (e.g. Image > Lookup Tables > Fire).

4. Morphological segmentation of optic vesicle tissues in Fiji/ImageJ.

“Segmentation > Segmentation Editor”

Prior to subsequent analysis, the particular tissue of interest (the optic vesicle) was distinguished from its neighbouring tissues (e.g. the closely associated cranial surface ectoderm and peri-ocular mesenchyme) through the process of manual segmentation. This involves generating a new 8-bit image stack in which each tissue or region of interest is represented by a unique numerical value, usually visualised by a unique colour. Fiji/ImageJ’s Segmentation Editor (Plugins > Segmentation > Segmentation Editor) is a convenient tool for efficiently segmenting multiple tissues of interest from a single image stack. The resulting ‘.label’ files are used both to project nuclear signalling activities onto the optic vesicle tissue morphology, and to perform non-rigid registration of data sets from different biological specimens.

As an aid to identifying closely associated tissue layers, it is useful to merge both the Propidium Iodide and AlexaFluor488 data channels, and particularly to increase the brightness of the AlexaFluor488 channel since the faint background auto-fluorescence of this channel can help to distinguish tissue boundaries. Moreover, it is also helpful to reduce the X and Y resolution of the merged image stack (known as down-sampling) by a factor of three (Image > Adjust… > Size…) since this reduces the memory requirements and computation time for interpolation of the selected segments (see below).

To perform segmentation, open the merged, down-sampled image stack, followed by the built-in Segmentation Editor (Plugins > Segmentation > Segmentation Editor). The editor allows you to select regions of interest in multiple stack slices, and to interpolate between

these slices to efficiently select regions that span multiple slices. In any given slice, a region can be selected using the Selection Brush tool from the Fiji/ImageJ toolbar (Right-click the 'Oval selections' tool, and choose 'Selection Brush Tool' from the pop-up menu; double click the tool icon to change the selection brush size). After selecting the region of interest in multiple stack slices, the individual selections are connected by interpolation (click the 'I' icon in the editor window). The interpolated selection can then be added to a region of interest (select a region of interest in the 'Labels' list an click the '+' icon). When finished, selecting ok will close the editor and open two image stacks: the original merged image stack and a second 8-bit '.label' stack, which should be saved. If the merged image stack was down-sampled then the '.label' stack can now be up-sampled to the original image size/resolution (Image > Adjust > Size…, set 'Interpolation:' to 'None').

5. Projection of signalling levels onto optic vesicle tissue morphology in Fiji/ImageJ.

"Atlas Toolkit > 2. Project to Segment Label"

In order to relate molecular patterning events with tissue morphology, it is necessary to be able to 'project' the former onto the latter. The Atlas Toolkit includes a plugin "2. Project to Segment Label", which automates this process and makes use of both the normalised fluorescent signal (Step 3) and a segmented '.label' file (Step 4). This plugin works by firstly isolating the tissue of interest from the normalised data stack (according to the supplied '.label' file, allowing the user to select from multiple labels when present). It then divides the isolated data segment into a voxel lattice of a specified size (the 'Voxel Size' parameter measured in pixels or µm depending on the calibration of the normalised data stack; 12 µm was used for this study). Each voxel is then assigned a value corresponding to the mean average signal level of its local neighbourhood (the 'Sample Radius' parameter measured in voxels; a radius of three voxels was used for this study). Non-nuclear pixels (which have a zero value) are excluded from averaging, but are assigned the same value as nuclear pixels within the same voxel. This local averaging smoothens local fluctuations and signal noise, and fills the segmented tissue with the locally averaged signal level. The resulting cubic lattice is then 'cropped' to the correct surface morphology using the supplied '.label' file.

6. Group-wise elastic registration of 3D segmented data sets in Fiji/ImageJ.

“Atlas Toolkit > 3. Label Registration 3D”

“Atlas Toolkit > 4. Apply Label Registration”

“Atlas Toolkit > 5. Merge Registered Volumes”

In order to compare molecular patterning or other quantities between independent specimens, it is necessary to first align (register) the 3D segmented tissues. This task is particularly difficult due to natural variation in tissue morphology at microscopic scales, difficulties in precise stage matching of specimens, and variation in specimen orientation

Supplementary Table 2 | Algorithm for “3. Label Registration 3D”

1:	For each segmented volume, *n*:
2:	Isolate segment of interest
3:	For each iteration, *i*:
4:	For $p = 3$ orthogonal planes (XY, YZ, ZX):
5:	For each segmented volume, *n*:
6:	Make 2D average intensity projection,
7:	For $s = n$ moving (source) projections:
8:	For $t = n$ fixed (target) projections:
9:	Run bUnwarpJ to perform pairwise 2D registration and generate a set of transformation coefficients, $C_{s,t}$
10:	Calculate the set of mean transformation coefficients, $C_{mean,s}$
11:	For *z* slices in segmented volume:
12:	Apply mean transformation coefficients, $C_{mean,s}$
13:	For each segmented volume, *n*:
14:	Re-slice segmented volume to achieve next orthogonal plane
15:	For each segmented volume, *n*:
16:	Write an ‘.ots’ file containing an ordered sequence of i x p sets of mean transformation coefficients, $C_{mean,s}$
17:	Write a ‘.tif’ file containing the registered volume
18:	Write a ‘consensus.tif’ file containing the intersection of *n* registered volumes

during imaging. Fiji/ImageJ includes a number of methods for registration of 2D images, but currently has no option for non-rigid (elastic) registration of 3D volumes.

A central feature of the Atlas Toolkit is the plugin "3. Label Registration 3D" which extends Fiji/ImageJ's built-in elastic 2D registration algorithm bUnwarpJ[1] to align two or more segmented volumes (from Step 4). Two or more '.label' files are supplied to the plugin, which then performs a series of 2D registrations (one for each orthogonal plane, e.g. XY, YZ, ZX), and this process can be repeated for multiple iterations until a satisfactory alignment is achieved.

Instead of the more usual approach of registering multiple 'moving' (source) images to a single 'fixed' (target) image, the plugin registers each individual volume to every volume in the data set (including itself), in order to calculate the mean average transformation. In this way, all images are registered to one-other and so converge together, yielding the 'consensus morphology' for the tissue of interest. This averaging is performed for each orthogonal plane and serves to minimise the degree of deformation imposed on each volume.

The "3. Label Registration 3D" algorithm is outlined in Supplementary Table 2. The output for the plugin includes a number of files: an '.ots' file (orthogonal transform sequence) describing how each '.label' file was transformed; a transformed version of each '.label'; a single 'consensus.tif' file representing the consensus morphology for the region of interest.

Once a set of '.label' files has been registered, the generated '.ots' files are used to transform the corresponding morphological projections from Step 5 (above) using the plugin "4. Apply Label Registration". Once each morphological projection has been transformed the data sets can be compared. For example, the utility plugin "5. Merge Registered Volumes" is used to calculate the mean and standard deviation from multiple volumes that contain independent observations of the same variable.

7. Hierarchical clustering of molecular patterning using Cluster3.0 and visualisation in Fiji/ImageJ.

"Atlas Toolkit > 6. Sample Volumes for Clustering"

"Atlas Toolkit > 7. Cluster Viewer"

The plugins “6. Sample Volumes for Clustering” and “7. Cluster Viewer” enable analysis of registered volumes by hierarchical clustering techniques using the open source clustering software Cluster3.0[5] (http://bonsai.hgc.jp/~mdehoon/software/cluster/).

The “6. Sample Volumes for Clustering” plugin accepts a number of registered data volumes (in the form of ‘.tif’ files). It divides the volumes into voxels of a given size (e.g. 18 μm (X) x 18 μm (Y) x 18 μm (Z) were used for this study) and samples the mean average signal level within each voxel. Empty pixels (those with a value of zero) are ignored, and the results are written as a table that should be saved as a text (‘.txt’) file, which serves as an input file for Cluster3.0.

To perform hierarchical clustering of this data, download and run Cluster3.0 (http://bonsai.hgc.jp/~mdehoon/software/cluster/). Use the “File > Open Data” menu option to select the ‘.txt’ file to be analysed. Under the ‘Hierarchical’ tab, in the ‘Arrays’ section, select the checkbox labelled “Cluster” and choose an option for ‘Similarity Metric’ (‘Euclidean Distance’ was selected for this study). Next, click a button to run the desired clustering method (‘Complete linkage’ was used for this study). Once finished, the software will write two output files to the same folder as the ‘.txt’ input file: the ‘.cdt’ file contains a table of the clustered data; the ‘.atr’ file describes the hierarchical tree.

The “7. Cluster Viewer” plugin performs 3D reconstruction and visualisation of clustered volumes. Moreover, the reconstructed results can be launched in the Fiji/ImageJ 3D Volume Viewer for generating movies. The plugin requires the ‘.cdt’ and ‘.atr’ files written by Cluster3.0, as well as a ‘consensus.tif’ file written by the “3. Label Registration 3D” plugin (Step 6, above). In addition to the 3D reconstruction, it generates a dendrogram representing a user-defined subset of the hierarchical tree (only the longest branches are shown in the present study), and a 2D heat-map representation of the mean quantities (e.g. nuclear protein levels in this study) within each cluster.

8. Numerical evaluation of Atlas Toolkit registration performance using a synthetic dataset.

Since real optic vesicle tissues lack objective landmarks, it was necessary to evaluate registration performance using a simulated dataset that was modified to include synthetic landmarks.

The segment label from a real Optic Vesicle object was augmented by addition of seven arbitrarily positioned spherical landmarks. The landmarks and segment label where then separated into two files, and both were identically deformed by translation, rotation and/or scaling, in order to create three manually deformed datasets. The three manually deformed segment labels were registered together using our tool “3. Label Registration 3D” for between one and six iterations. The resulting ‘.ots’ files were then used to transform the three corresponding landmark files into the shared coordinate system of the consensus object. Following landmark transformation, the Fiji/ImageJ 3D Object Counter function was used to locate the centres of the spherical landmarks, and the pair-wise distances between corresponding landmarks were calculated (Fig. 1f – h; Supp. Fig. S1a). The mean pair-wise distance between all corresponding landmark pairs following one iteration was 6.12 μm +/- 4.78 μm (mean +/- standard deviation), or 3.24 μm +/- 3.6 μm (mean +/- standard deviation) following six iterations.

In addition to landmark distances, we also measured the mean volumetric overlap between all three optic vesicles and their shared consensus morphology (Supplementary Fig. S1b). For one iteration, the mean volumetric overlap was 93.26 % +/- 0.19 % (mean +/- standard deviation), and for six iterations it was 95.85 % +/- 0.14 % (mean +/- standard deviation).

The time required to compute these registrations was 180 seconds (for one iteration and 1,680 seconds for six iterations (Supp. Fig. S1c), on a MacBook Pro laptop computer with 2.7 GHz Intel Core i7 CPU and 16 GB of RAM, running on battery power.

9. Numerical comparison of Atlas Toolkit with two BrainAligner methods.

In order to better gauge the performance of our landmark-free method, we used the same simulated dataset (described above) to evaluate the performance of a previously published tool, BrainAligner (http://penglab.janelia.org/proj/brainaligner/), which performs 3D pair-wise elastic registration in a landmark-dependent fashion. Because BrainAligner is a pair-wise

method, one of the volumes must be selected as a 'fixed' reference against which the other two volumes are registered. To avoid biasing the results by registering to any one specimen, all registrations were repeated with each specimen serving as 'fixed' reference and the results were averaged. For this exercise we compared two different BrainAligner methods.

For 'Method A', the BrainAligner tool was provided with the precise coordinates of all seven simulated landmarks in each of the three manually deformed optic vesicles. Since BrainAligner uses this landmark information to achieve its registration it should be expected to perform very well in this test. Indeed, for BrainAligner 'Method A', the mean distance between landmarks was 0.4 μm +/- 0.31 μm (mean +/- standard deviation; Supp. Fig. S1a), while volumetric overlap was 98.8 % +/- 0.3 % (mean +/- standard deviation; Supp. Fig. S1b). Thus BrainAligner 'Method A' is around eight times more accurate in registering landmarks than Atlas Toolkit, while the computation time for this method was comparable to Atlas Toolkit, taking 238 seconds on the MacBook Pro (Supp. Fig. S1c).

Many embryonic tissues and organs (including optic vesicles) do not contain multiple objective landmarks, and we wished to test the performance of BrainAligner in this more common situation. For 'Method B', precise landmark positions were supplied only for the 'fixed' optic vesicle. For the two 'moving' optic vesicles, precise landmark information was not provided. Instead we relied on BrainAligner's built-in landmark finding function to identify the corresponding landmarks in the two 'moving' datasets. This represents a situation in which the user chooses arbitrary landmark points in the 'fixed' dataset, allowing the software to automatically identify corresponding points in all other datasets.

For 'Method B', the mean distance between landmarks was 24.27 μm +/- 15.51 μm (mean +/- standard deviation; Supp. Fig. S1a), volumetric overlap was 62.98 % +/- 9.72 % (mean +/- standard deviation; Supp. Fig. S1b), and computation time was 277 seconds on the MacBook Pro (Supp. Fig. S1c). In this situation BrainAligner is almost 10 times less accurate than Atlas Toolkit. This is possibly because the 3D datasets undergoing registration contain purely binary information: pixel values indicate the presence or absence of tissue at each position in the volume. It is likely there is insufficient information for BrainAligner to accurately identify corresponding landmark features between the different 3D datasets.

10. Numerical comparison of Atlas Toolkit with the Computational Morphometry Toolkit (CMTK).

Since BrainAligner is intended for 3D pair-wise landmark-based registrations, whereas Atlas Toolkit is intended for 3D group-wise landmark-independent registrations, we compared our method's performance with another 3D group-wise landmark-independent registration tool included in the Computational Morphometry Toolkit (CMTK) – 'groupwise_warp' (http://www.nitrc.org/projects/cmtk).

'groupwise_warp' employs a true 3D algorithm to calculate 3D elastic deformations. This is in contrast to Atlas Toolkit, which decomposes the 3D registration problem into a series of orthogonal 2D elastic registrations. Consequently, 'groupwise_warp' might be expected to generate more accurate registrations at the cost of greatly increased computation time. We therefore tested it on the same synthetic dataset used to evaluate Atlas Toolkit and BrainAligner.

For 'groupwise_warp', the mean distance between landmarks was 10.85 μm +/- 9.35 μm (mean +/- standard deviation; Supp. Fig. 1a) and volumetric overlap was 99.52 % +/- 0.02 % (mean +/- standard deviation; Supp. Fig. 1b). 'groupwise_warp' did not run to completion on the MacBook Pro because the tool's memory requirements exceeded the 16 GB of available RAM. Instead this tool was run using a Windows 7 PC with 2.4 GHz Intel Xeon processor and 64 GB of RAM. The computation time on this machine was 152,895 seconds (around 42 hours; Supp. Fig. 1c).

The CMTK toolkit out-performed Atlas Toolkit and was comparable to BrainAligner 'Method A' in terms of volumetric overlap (Supp. Fig. 1b). However, in terms of landmark accuracy it was the second-worst method tested, being out-performed by both BrainAligner 'Method A' and Atlas Toolkit (one or six iterations; Supp. Fig. 1a). Moreover, BrainAligner 'Method A' and Atlas Toolkit (one iteration) were both around 1,000 times faster (Supp. Fig. S1c).

As with BrainAligner 'Method B', the inability of CMTK to accurately align landmarks as compared with Atlas Toolkit might again be explained by the lack of suitable information in the binary (black/white) 3D datasets. Indeed, the best information available to the CMTK tool is likely to be the transition between 'white' and 'black' pixels at the surface interface between

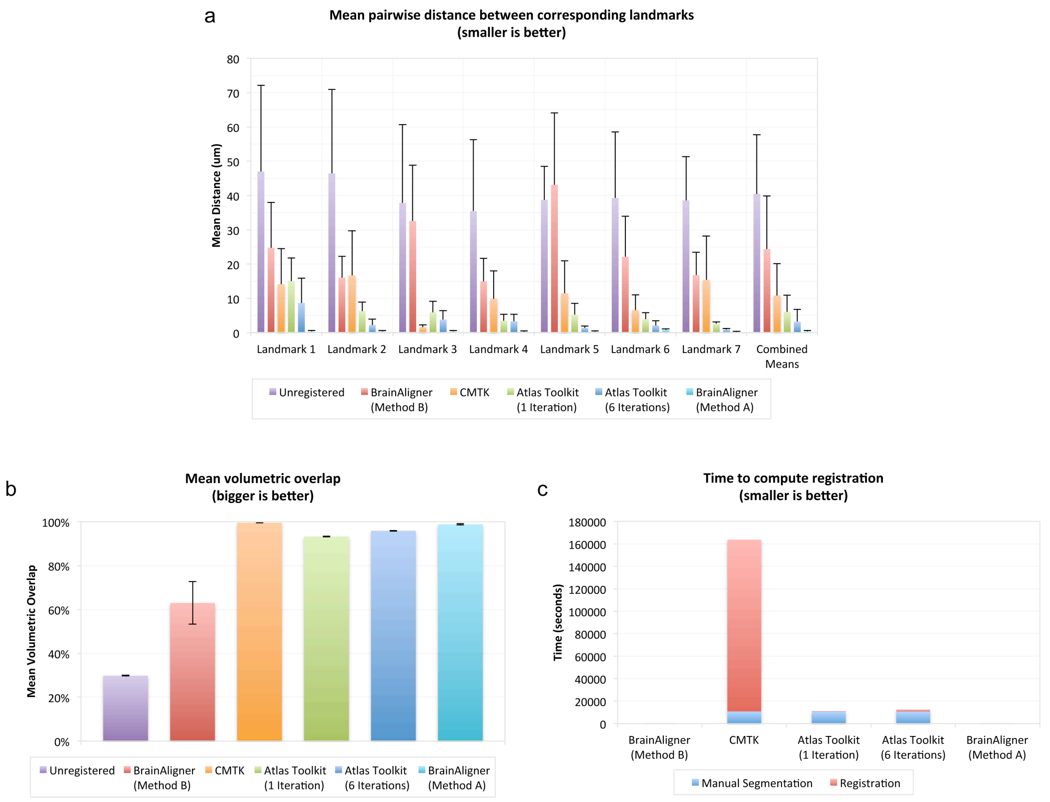

Supplementary Figure S1 | Numerical evaluation of Atlas Toolkit in comparison with BrainAligner and Computation Morphometry Toolkit (CMTK). A simulated dataset comprising three manually deformed Optic Vesicles containing seven simulated landmarks (Fig. 1f – h; described in Supplementary Methods) was used to numerically evaluate the performance of five 3D registration methods: Atlas Toolkit with one iteration; Atlas Toolkit with 6 iterations; the CMTK tool 'groupwise_warp'; pair-wise BrainAligner 'Method A' using landmarks accurately specified for all three Optic Vesicles; pair-wise BrainAligner 'Method B' with landmarks specified for the 'fixed' Optic Vesicle and auto-detecting landmarks for the two 'moving' Optic Vesicles. For each of the five methods we measured: **(a)** Mean pair-wise distance between the seven individual landmarks, and the combined average distance for all landmarks. **(b)** Mean volumetric overlap between all three Optic Vesicles. **(c)** Time to compute Optic Vesicle registrations, including manual segmentation time for landmark-free methods (estimated at 180 minutes). See supplementary methods for full details. Error bars indicate standard deviations.

optic vesicle and empty space. This could explain both the excellent volumetric overlap score and the inferior internal landmark correspondence observed with CMTK. It may be that the

superior landmark correspondences observed with Atlas Toolkit (versus CMTK 'groupwise_warp' and BrainAligner 'Method B') are due to the conversion of information-poor binary 3D datasets to information-rich 2D grey-scale average intensity projections.

Known Limitations

We have used Atlas Toolkit to successfully register various 'thick' epithelial tissue morphologies including optic vesicle tissues from HH10 – HH14, and optic cup (Supp. Fig. S2a – c) and lens vesicle tissues (Supp. Fig. S2d – f) from HH16 chick embryos. However, a consequence of defining 'consensus morphology' as the intersection of all registered objects is that imperfect registration causes 'erosion' of the consensus morphology (schematised in Supp. Fig. S2g – j). Whereas 'thick' columnar or (pseudo-) stratified epithelia (e.g. HH16 optic cup, Supp. Fig. S2a – c; HH16 lens vesicle, Supp. Fig. S2d – f; HH10 & HH12 optic vesicles, main study) are more tolerant of small local mismatches between the registered objects (schematised in Supp. Fig. S2g & h), the thin and undulating morphologies of squamous epithelia often fail to achieve good intersections (e.g. HH16 surface ectoderm, Supp. Fig. S2d – f; schematised in Supp. Fig. S2i & j). This failure to register squamous epithelia appears to arise as the tissue's smallest dimension approaches the magnitude of registration error produced by Atlas Toolkit (Fig. 1f; Supp. Fig. S1a). The test data included with Supplementary Software 1 provides examples of both success (HH10 optic vesicle; label channel 1) and fail cases (HH10 surface ectoderm; label channel 2) within the same datasets.

Manual segmentation of entire objects from raw datasets is more time-consuming than placing a small number of precise fiduciary markers. It will also likely require training of personnel to accurately distinguish closely associated tissues (e.g. surface ectoderm versus optic vesicle epithelia in the developing eye) in the absence of molecular labels, and especially where optical sectioning is oblique to the junction between tissues. However, fiduciary marker placement depends upon the presence of user-identifiable landmarks, but not all tissues are rich in small landmark features (e.g. the optic vesicle, limb bud, heart tube). Thus, segmentation-guided registration as provided by Atlas Toolkit may be preferable for landmark-poor tissues.

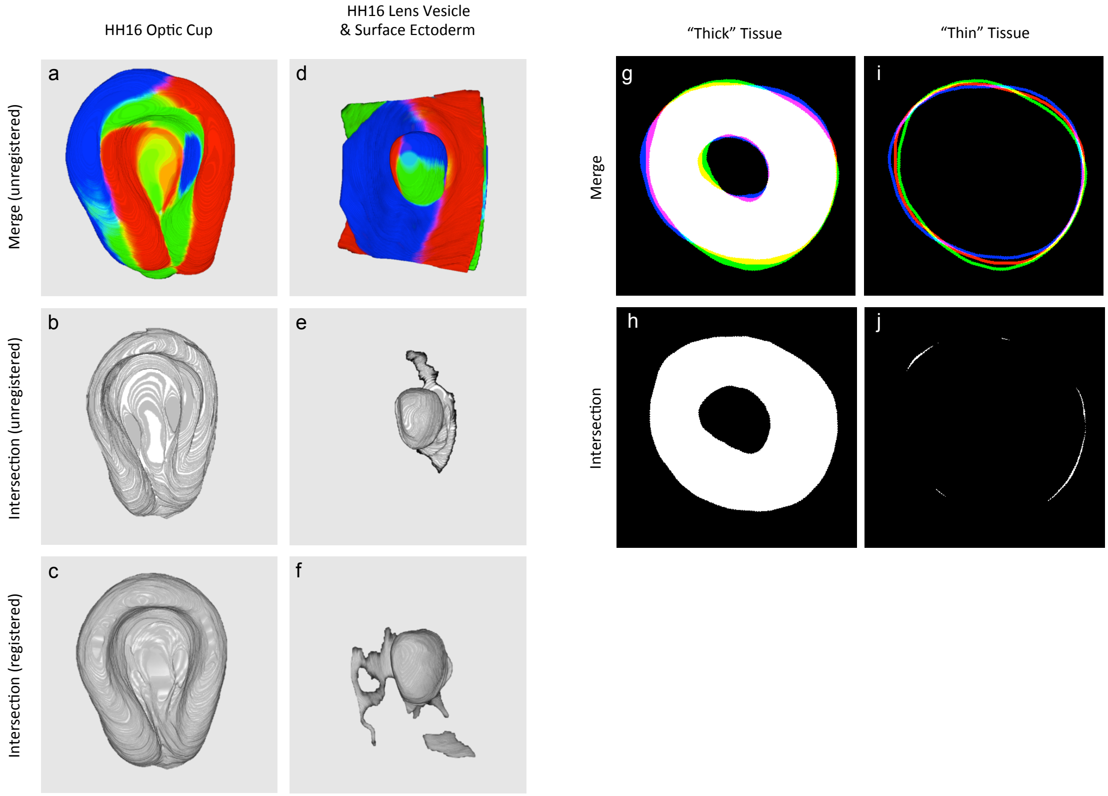

Supplementary Figure S2 | Further examples of success and fail cases. **(a – c)** HH16 chick Optic Cups. **(a)** 3D reconstruction of three superimposed Optic Cup tissues (red, green, blue) segmented from HH16 chick embryos before group-wise 3D registration. **(b)** Intersection of the three Optic Cups shown in (b) before group-wise 3D registration. **(c)** Intersection of the three Optic Cups shown in (b) after group-wise 3D registration using Atlas Toolkit (one iteration). **(d – f)** HH16 chick Lens Vesicles with surrounding Surface Ectoderm. **(d)** 3D reconstruction of three superimposed Lens Vesicles and surrounding Surface Ectoderm tissues (red, green, blue) before group-wise 3D registration. **(e)** Intersection of the three Lens Vesicle/Surface Ectoderm tissues shown in (d) before group-wise 3D registration. **(f)** Intersection of the three Lens Vesicle/Surface Ectoderm tissues shown in (d) after group-wise 3D registration with Atlas Toolkit (four iterations). **(g – j)** Schematic representations of consensus morphology 'erosion' caused by imperfect alignments. **(g)** Imperfect alignment of three superimposed 'thick' tissues (red, green, blue). **(h)** Intersection of the three 'thick' tissues shown in (g). **(i)** Imperfect alignment of three superimposed 'thin' tissues (red, green, blue). The alignment error is the same as for (g). **(j)** Intersection of the three 'thin' tissues shown in (i).

References

1. Hamburger, V. & Hamilton, H.L. *J. Morph.* 88, 49-92 (1951).
2. Hama, H. et al. *Nat. Neurosci.* 14, 1481-1488 (2011).
3. Arganda-Carreras, I. *et al. Lecture Notes in Computer Science* **4241**, 85-95 (Springer, 2006).
4. Schohl, A. & Fagotto, F. *Development* 129, 37-52 (2002).
5. de Hoon, M. J. L. *et al. Bioinformatics* **20**, 1453-1454 (2004).
